# Nephron deficit and low podocyte density increase risk of albuminuria and glomerulosclerosis in a model of diabetes

**DOI:** 10.14814/phy2.15579

**Published:** 2023-01-25

**Authors:** Sarah E. Gazzard, James van der Wolde, Kotaro Haruhara, John F. Bertram, Luise A. Cullen‐McEwen

**Affiliations:** ^1^ Department of Anatomy and Developmental Biology, Monash Biomedicine Discovery Institute Monash University Melbourne Australia; ^2^ Division of Nephrology and Hypertension, Department of Internal Medicine The Jikei University School of Medicine Tokyo Japan; ^3^ ARC Training Centre for Cell and Tissue Engineering Technologies Melbourne Australia

**Keywords:** developmental programming, diabetes, hyperglycemia, kidney, nephron, podocyte endowment

## Abstract

Podocytes are terminally differentiated epithelial cells in glomeruli. Podocyte injury and loss are features of many diseases leading to chronic kidney disease (CKD). The developmental origins of health and disease hypothesis propose an adverse intrauterine environment can lead to CKD later in life, especially when a second postnatal challenge is experienced. The aim of this study was to examine whether a suboptimal maternal environment would result in reduced podocyte endowment, increasing susceptibility to diabetes‐induced renal injury. Female C57BL/6 mice were fed a low protein diet (LPD) to induce growth restriction or a normal protein diet (NPD) from 3 weeks before mating until weaning (postnatal Day 21, P21) when nephron and podocyte endowment were assessed in one male and one female offspring per litter. Littermates were administered streptozotocin or vehicle at 6 weeks of age. Urinary albumin excretion, glomerular size, and podometrics were assessed following 18 weeks of hyperglycemia. LPD offspring were growth restricted and had lower nephron and podocyte number at P21. However, by 24 weeks the podocyte deficit was no longer evident and despite low nephron endowment neither albuminuria nor glomerulosclerosis were observed. Podocyte number was unaffected by 18 weeks of hyperglycemia in NPD and LPD offspring. Diabetes increased glomerular volume reducing podocyte density, with more pronounced effects in LPD offspring. LPD and NPD diabetic offspring developed mild albuminuria with LPD demonstrating an earlier onset. LPD offspring also developed glomerular pathology. These findings indicate that growth‐restricted LPD offspring with low nephron number and normalized podocyte endowment were more susceptible to alterations in glomerular volume and podocyte density leading to more rapid onset of albuminuria and renal injury than NPD offspring.

## Graphical abstract

This study examined whether a suboptimal maternal environment (maternal low protein diet) would result in low podocyte endowment and increase the risk of diabetes‐induced renal injury. The present findings demonstrate that a permanent nephron deficit combined with a postnatal hyperglycemic challenge can increase the risk of albuminuria and glomerulosclerosis. These changes occur in conjunction with glomerular hypertrophy and relative podocyte depletion providing new insights into the associations between birth weight, podocyte indexes, and glomerular pathology.

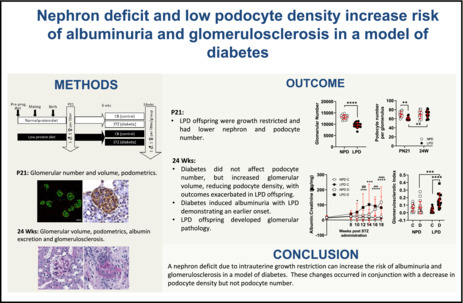

## 1 INTRODUCTION

Diabetes is the leading cause of chronic kidney disease (CKD) (Alicic et al., 2017). A dramatic rise in the prevalence of diabetes has escalated the number of CKD patients, with CKD now affecting 9.1% of the world's population (Collaboration GCKD, 2020). Despite therapeutic strategies to manage diabetes a significant portion of diabetic patients still end up with kidney failure (Costacou & Orchard, 2018; Zou et al., 2022).

In the last 30 years, a clear association between low birth weight/intrauterine growth restriction and increased risk of adult kidney disease (Hoy et al., 1999; Vikse et al., 2008; White et al., 2009) has resulted in a multitude of studies assessing the impact of the maternal environment on offspring nephron endowment (number of nephrons at the completion of nephrogenesis) and the resulting likelihood of kidney disease as an adult (Gray et al., 2010; Hoppe, Evans, Moritz, et al., 2007; Langley‐Evans et al., 1999; Singh et al., 2007; Villar‐Martini et al., 2009; Walton et al., 2017). While it is now well accepted that low birth weight/intrauterine growth restriction is associated with a permanent low nephron endowment we have recently shown that growth restriction, induced by low protein diet, also impacts podocyte endowment (Cullen‐McEwen et al., 2021).

Podocytes are terminally differentiated epithelial cells of glomeruli. Human and animal studies have shown podocyte loss in association with aging, hypertension, and diabetes leads to proteinuria, glomerulosclerosis, and loss of renal function (Dalla Vestra et al., 2003; Hodgin et al., 2015; Pagtalunan et al., 1997; Puelles et al., 2019; Puelles, van der Wolde, et al., 2016; Steffes et al., 2001; Wang et al., 2009; Wharram et al., 2005; Wiggins et al., 2005). Given the importance of podocyte number and density for glomerular health and the link between diabetes and podocyte depletion, we hypothesized that growth‐restricted mice with a congenital nephron deficit would also have a congenital podocyte deficit and that the induction of hyperglycemia in early adulthood would further deplete podocyte number placing these offspring at greater susceptibility to develop albuminuria and glomerulosclerosis in adulthood.

In this study, we used a model of maternal protein restriction during pregnancy and lactation, known to induce growth restriction and a 20–30% nephron deficit (Hoppe, Evans, Bertram, et al., 2007; Langley‐Evans et al., 1999). We found growth‐restricted offspring had low podocyte endowment at weaning, but podocyte number had normalized by 6 months of age and despite a low nephron endowment, growth‐restricted offspring had normal kidney function in young adulthood. Hyperglycemia did not alter podocyte number in either growth‐restricted or normal birth weight offspring but did induce glomerular hypertrophy and a decrease in podocyte density (relative podocyte depletion) with growth‐restricted offspring more susceptible than normal offspring, leading to earlier onset albuminuria and pathology than normal offspring.

## 2 MATERIALS AND METHODS

### 2.1 Animals and diets

**FIGURE 1:**
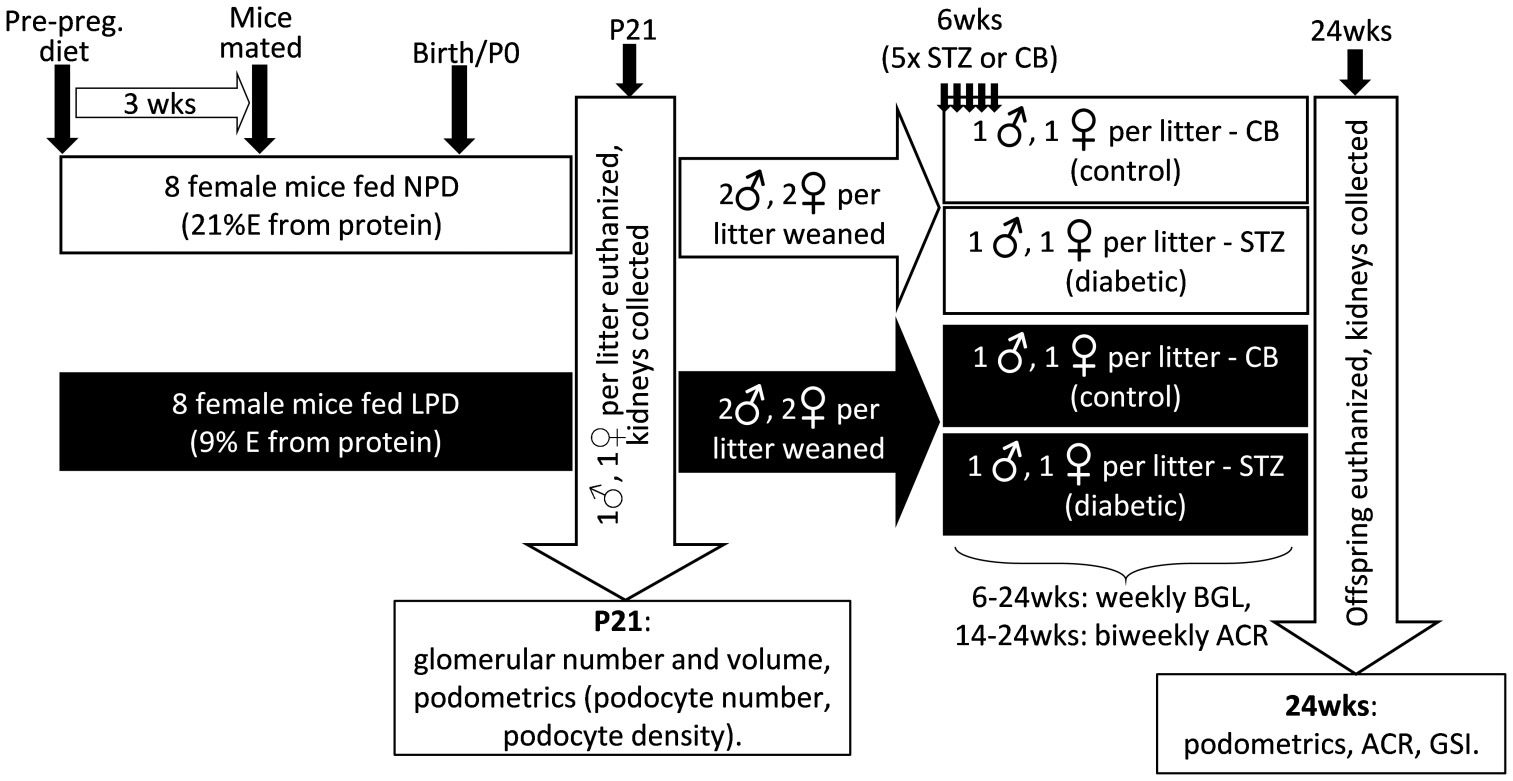
Schematic of experimental design. Female C57Bl6/J mice were fed a normal protein diet (NPD; *n* = 8) or a low protein diet (LPD; *n* = 8) from 3 weeks prior to mating and up to weaning at P21. One male and one female offspring per litter was euthanized at P21 (weaning) for assessment of glomerular and podocyte indices. Two male and two female offspring per litter were weaned onto standard laboratory chow. At 6 weeks of age, one male and one female per litter were administered i.p. injections of streptozotocin (55 mg/kg) to induce diabetes or citrate buffer (control); daily over 5 days. P, protein; F, fat; STZ, streptozotocin; CB, citrate buffer; ACR, albumin creatinine ratio; GSI, glomerulosclerotic index; BGL, blood glucose level; 6 W, 6 weeks of age; 24 W, 24 weeks of age.

All experiments were conducted in accordance with guidelines set by the Monash University Animals Ethics Committee (ethics approval number: MARP/2016/162). The experimental design is shown in Figure 1. Briefly, 8–10‐week‐old C57Bl6/J dams were fed ad libitum a low protein diet (LPD; 9% digestible energy from protein, 16.2 MJ/kg; SF01‐026, Specialty Feeds, Glen Forrest, Australia) or an isocaloric normal protein diet (NPD; 21% digestible energy from protein, 16.1 MJ/kg; AIN93G, Specialty Feeds, Glen Forrest, Australia) 3 weeks prior to mating, throughout pregnancy and lactation. Dietary compositions are shown in Table 1.

**TABLE 1:** Diet composition

Specialty feeds code	NPD	LPD
	AIN93G	SF01‐026: 8% low protein modification of AIN93G
	Calculated nutritional parameters	
Protein	19.4%	8.4%
Fat	7%	7%
Crude fiber	4.7%	4.7%
Adequate dietary fiber	4.7%	4.7%
Digestible energy	16.1 MJ/kg	16.2 MJ/kg
% Total calculated digestible energy from lipids	16%	16%
% Total calculated digestible energy from protein	21%	9%
	Ingredients (g/kg)	
Casein (Acid)	200	87
Sucrose	100	200
Canola oil	70	70
Cellulose	50	50
Wheat starch	404	417
DL Methionine	3.0	3.0
Calcium carbonate	13.1	13.1
Sodium chloride	2.6	2.6
AIN93 trace minerals	1.4	1.4
Potassium citrate	2.5	2.5
Potassium dihydrogen phosphate	6.9	6.9
Potassium sulphate	1.6	1.6
Choline chloride (75%)	2.5	2.5
AIN93 vitamins	10	10

To control for litter size across the two groups, litters with <4 or >8 pups were excluded from analysis. At postnatal Day 21 (P21), one male and one female pup per litter were euthanized, and kidneys removed for nephron and podocyte morphometrics (*n* = 8 litters per diet). Remaining offspring were weaned onto standard laboratory chow (Barastoc; 9% fat, 22% protein; 13.2 MJ/kg) from P21. At 6 weeks of age, offspring were injected with either streptozotocin (STZ) to induce diabetes or volume‐matched citrate buffer. STZ was administered daily at 55 mg/kg via intraperitoneal injection following a 5‐h fast over 5 consecutive days. Animals that failed to reach 18 mmoL/L blood glucose 3 weeks after STZ administration were excluded from analysis. At all times, mice had ad libitum access to food and water until tissue collection at 24 weeks of age (Figure 1).

### 2.2 Perfusion fixation and tissue collection

At P21 and 24 weeks, mice were anesthetized using 100% isoflurane and euthanized by exsanguination. An incision was made along the thoracic wall and through the diaphragm and ribcage to expose the heart. The incision was then extended along the abdominal wall to expose the kidneys and vena cava. A winged infusion set with a 25‐gauge (P21) or 23‐gauge butterfly needle (24 weeks) connected to a 50‐ml syringe was inserted into the left ventricle and the inferior vena cava proximal to the kidneys cut. Kidneys were cleared of blood by infusion of 20 ml of phosphate‐buffered saline (PBS) at 5 ml/min through the left ventricle. Kidneys were fixed by perfusion of 20 ml of formalin administered at 5 ml/min. All perfusions were performed by one experienced technician to limit variation between mice. Both kidneys were immersion‐fixed in 10% formalin. Right kidneys were sliced with a series of razor blades evenly spaced at 800 μm and one 800 μm mid‐hilar slice was selected for immunofluorescence. Whole left kidneys were embedded in paraffin and processed for assessment of glomerular number. Glomeruli were not counted at 24 weeks.

### 2.3 Glomerular number

Total glomerular number, and thereby total nephron number, per kidney was estimated at P21 using the physical disector/fractionator combination. In brief, left kidneys embedded in paraffin were exhaustively sectioned at 5 μm. Ten evenly spaced section pairs were systematically sampled and stained with lectin peanut agglutinin (PNA; Sigma‐Aldrich, Castle Hill, NSW, Australia; L3165) to identify the plasma membrane of podocytes and counterstained with hematoxylin. Section pairs were projected using a light microscope, and all PNA‐positive glomeruli were counted using the disector counting principle (Cullen‐McEwen, Armitage, et al., 2012; Cullen‐McEwen, Douglas‐Denton, et al., 2012).

### 2.4 Immunofluorescence and imaging

At P21 and 24 weeks, podocytes were counted in 20 whole glomeruli in a single 800‐μm slice from each mouse podocytes were identified by their nuclear expression of p57 (sc‐8298; Santa Cruz Biotechnology, Santa Cruz, CA) and cytoplasmic expression of synaptopodin (sc‐21,537; Santa Cruz Biotechnology). Following immunofluorescence labeling kidney slices were cleared using ethyl cinnamate (ECi; 99% concentration, product number 112372; Sigma‐Aldrich) and then imaged using a Leica SP8 Multiphoton Microscope fitted with a Leica 20× BABB objective lens. Twenty glomeruli per kidney were imaged over the z‐axis at 1‐μm intervals to obtain a stack of optical sections through each whole glomerulus.

### 2.5 Podocyte number, glomerular volume, and podocyte density

**FIGURE 2:**
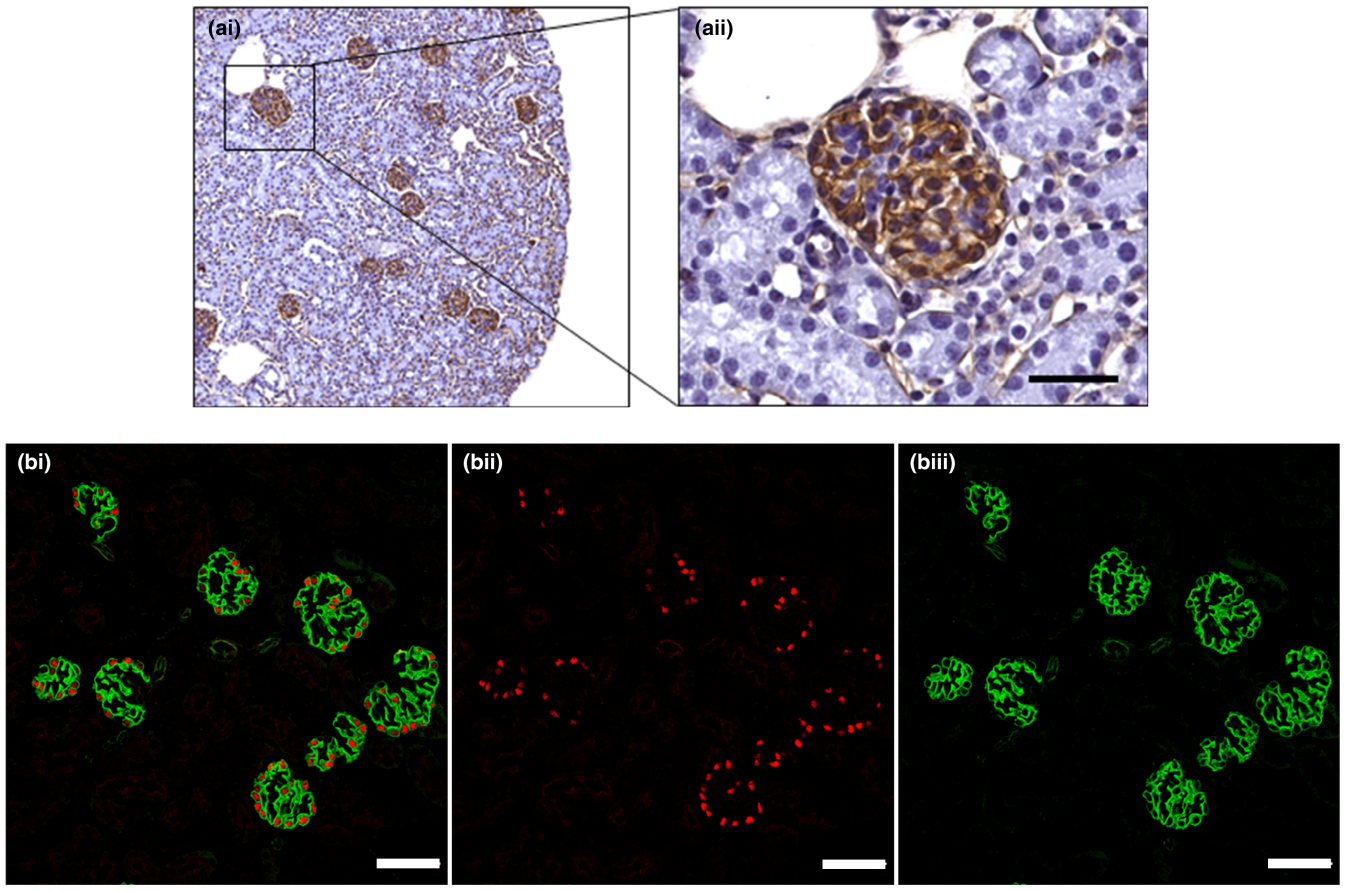
Glomerular and podocyte labelling. (a) Representative peanut agglutinin (PNA) lectin histochemical labelling of glomeruli for stereological assessment of glomerular number at P21; scale bar = 50 μm. (b) Representative double immunofluorescence labelling (i) to identify podocyte nuclei (ii; p57 positive) and podocyte cytoplasm (iii; synaptopodin positive). Such sections through whole glomeruli were used to quantify glomerular volume, podocyte number per glomerulus and podocyte density at P21 and 24 weeks of age. Scale bar = 50 μm.

Podocytes were defined as cells with a p57^+^ nucleus and SNP^+^ cytoplasm. Figure 2 shows optical sections from a glomerulus labeled with synaptopodin and p57. The volume of each of the 20 whole sampled glomeruli per mouse used for podocyte counting was estimated using the Cavalieri principle (Gundersen et al., 1988). In brief, with a random start, the area of every 7th optical section per glomerular tuft was measured using Fiji imaging software. Glomerular volume was calculated by multiplying the sum of the section areas per glomerulus by the thickness of the optical sections, by the reciprocal of the section sampling fraction. Podocyte density was calculated by dividing total podocyte number for each glomerulus by the volume of that individual glomerulus.

### 2.6 Blood glucose and albumin: Creatinine ratios

Blood glucose was measured in offspring at 6 weeks (baseline) and weekly thereafter until tissue collection at 24 weeks of age via a tail vein prick and a glucometer test after a 5‐h fast. Morning spot urine was collected at the same time points for analysis of albumin: creatinine ratio. Albumin levels were analyzed using direct competitive ELISA (Exocell; Albuwell, Philadelphia, USA). Creatinine was measured using the Jaffe’ reaction of alkaline picrate with creatinine (Exocell).

### 2.7 Histopathology

**FIGURE 3:**
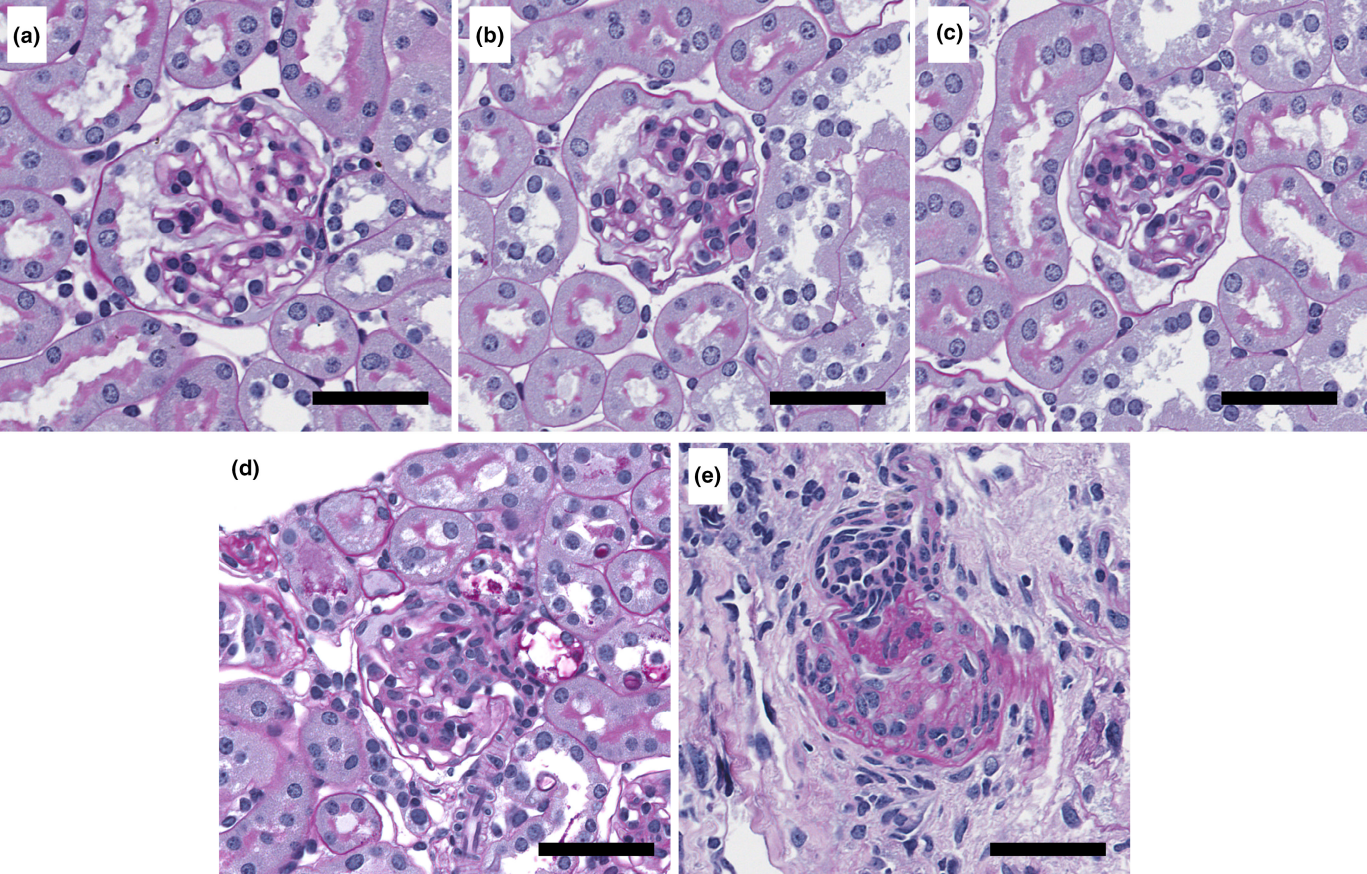
Scoring glomerulosclerotic index at 24 weeks in control and diabetic mice. One mid‐hilar 2 μm section from each kidney at 24 weeks was stained with periodic acid Schiff (PAS). Every glomerulus in the section was scored for sclerosis to calculate a glomerulosclerosis index (GSI). A score of 0 was assigned to normal glomeruli (a), a score of 1 if sclerosis was present in up to 25% of the glomerulus (b), a score of 2 if sclerosis was present in 26%–50% of the glomerulus (c), a score of 3 if sclerosis was present in 51% to 75% of the glomerulus (d), and a score of 4 if sclerosis was present in 76% to 100% of the glomerulus (e).

One mid‐hilar 2‐μm section from each kidney at 24 weeks was stained with periodic acid Schiff (PAS) and imaged with Leica Aperio AT Turbo for assessment of renal histopathology. Every glomerulus was scored for sclerosis to calculate a glomerulosclerosis index (GSI). A score of 0 was assigned to normal glomeruli (Figure 3a), a score of 1 if sclerosis was present in up to 25% of the glomerulus (Figure 3b), a score of 2 if sclerosis was present in 26%–50% of the glomerulus (Figure 3c), a score of 3 if sclerosis was present in 51%–75% of the glomerulus (Figure 3d), and a score of 4 if sclerosis was present in 76%–100% of the glomerulus (Figure 3e). GSI was calculated using the formula (Cahill et al., 1996):

$$\mathrm{GSI}=\frac{\left[\left(1\times N1\right)+\left(2\times N2\right)+\left(3\times N3\right)+\left(4\times N4\right)\right]}{\left(N0+N1+N2+N3+N4\right)}$$

where *N* is the number of glomeruli with each given score for a given section.

### 2.8 Statistical analysis

One male and one female offspring for each of 8 NPD and 8 LPD litters were studied at P21 (*n* = 32 mice at P21). Preweaning bodyweight, glomerular number per kidney, glomerular number per gram of bodyweight, and kidney weight were analyzed by two‐way ANOVA (diet and sex). To examine the effect of growth restriction and low nephron endowment on podocyte and glomerular indices across time, podocyte number per glomerulus, podocyte density and glomerular volume at P21 and 24 weeks were analyzed by three‐way ANOVA (diet, sex and time). To analyze the effects of growth restriction and low nephron endowment on susceptibility to adult diabetic kidney disease, four groups of mice were examined (NPD/control, NPD/diabetic, LPD/control and LPD/diabetic; Figure 1) with 1 male and one female for each diet and each treatment group (*n* = 64 mice at 24 weeks). ACR data in diabetic mice were not normally distributed, so values were log transformed prior to analysis. Data at 24 weeks were analyzed by three‐way ANOVA (diet, sex, and treatment). In all cases, when no sex difference was identified data were consolidated. Given the obvious sex difference in blood glucose following STZ administration, male and female blood glucose profiles over the experimental period were analyzed separately using three‐way ANOVA (diet, treatment, and time). Statistical analyses were performed utilizing GraphPad Prism 9. Data are presented as mean ± SD; *n* refers to number of litters. *p*‐values presented have been adjusted for multiple comparisons using Sidak correction. Statistical significance was defined as *p* < 0.05.

## 3 RESULTS

### 3.1 Maternal LPD induced low birth weight and low nephron number

Litters with 4–8 pups were selected for analysis. Litter size data are presented in Table 2. Preweaning bodyweights, kidney weight, kidney volume, and nephron number were similar in male and female offspring (Table 2).

**TABLE 2:** Summary of morphometric data at postnatal days 2 (P2), 7 (P7), 14 (P14), and 21 (P21)

Maternal diet	NPD		LPD											
Litter size	6.13 ± 1.81		6.00 ± 1.31		Two‐way ANOVA			Three‐way ANOVA						
	NPD (male)	NPD (female)	LPD (male)	LPD (female)	*p*_diet_	*p*_sex_	*p*_diet*sex_	*p*_diet_	*p*_sex_	*p*_time_	*p*_diet*sex_	*p*_time*diet_	*p*_time*sex_	*p*_diet*time*sex_
Bodyweight P2 (g)	1.50 ± 0.31	1.52 ± 0.20	1.38 ± 0.09	1.31 ± 0.10	**0.0006**	0.564	0.358							
Bodyweight P7 (g)	3.58 ± 0.78	3.60 ± 0.60	2.77 ± 0.20	2.53 ± 0.27	**<0.0001**	0.567	0.495							
Bodyweight P14 (g)	6.70 ± 0.56	6.29 ± 0.53	4.63 ± 0.46	4.45 ± 0.63	**<0.0001**	0.088	0.516							
Bodyweight P21 (g)	9.38 ± 0.85	9.11 ± 0.70	6.79 ± 0.76	6.74 ± 0.68	**<0.0001**	0.073	0.196							
Total kidney weight (g)	0.20 ± 0.02	0.19 ± 0.02	0.14 ± 0.01	0.14 ± 0.02	**<0.0001**	0.280	0.303							
Kidney volume (mm^3^)	41.9 ± 2.9	38.4 ± 6.6	25.7 ± 2.9	26.2 ± 5.1	**<0.0001**	0.371	0.228							
Nephron number	13,449 ± 869	12,833 ± 1022	9606 ± 900	9450 ± 1422	**<0.0001**	0.319	0.550							
Nephron number/gBW	1434 ± 55	1418 ± 150	1415 ± 97	1414 ± 182	0.813	0.861	0.866							
Podocyte number /glomerulus (P21)	72.2 ± 5.9	67.3 ± 5.3	61.6 ± 3.8	60.4 ± 4.7				**0.04**	0.17	**0.03**	0.05	**0.004**	0.67	0.37
Podocyte number /glomerulus (24 W)	71.8 ± 7.3	65.2 ± 8.8	68.3 ± 7.3	71.6 ± 8.9										
Glomerular volume (P21) ×10^5^ μm^3^	1.44 ± 0.11	1.44 ± 0.16	1.11 ± 0.06	1.23 ± 0.10				**0.006**	0.97	**<0.0001**	0.96	0.18	0.42	0.42
Glomerular volume (24 W) ×10^5^ μm^3^	2.40 ± 0.34	2.40 ± 0.39	2.35 ± 0.36	2.25 ± 0.24										
Podocyte density (P21; number per 10^5^ μm^3^)	51.9 ± 5.8	48.5 ± 4.1	56.6 ± 3.1	51.9 ± 3.6				**0.009**	0.06	**<0.0001**	0.40	0.28	0.07	0.15
Podocyte density (24 W; number per 10^5^ μm^3^)	30.6 ± 3.1	28.1 ± 5.6	29.8 ± 4.7	32.2 ± 2.9										

*Note*: Values are mean ± SD. Analyzed by two‐way ANOVA for sex and diet, and three‐way ANOVA for sex, diet, and time with significant differences (*p* < 0.05) highlighted in bold.

**FIGURE 4:**
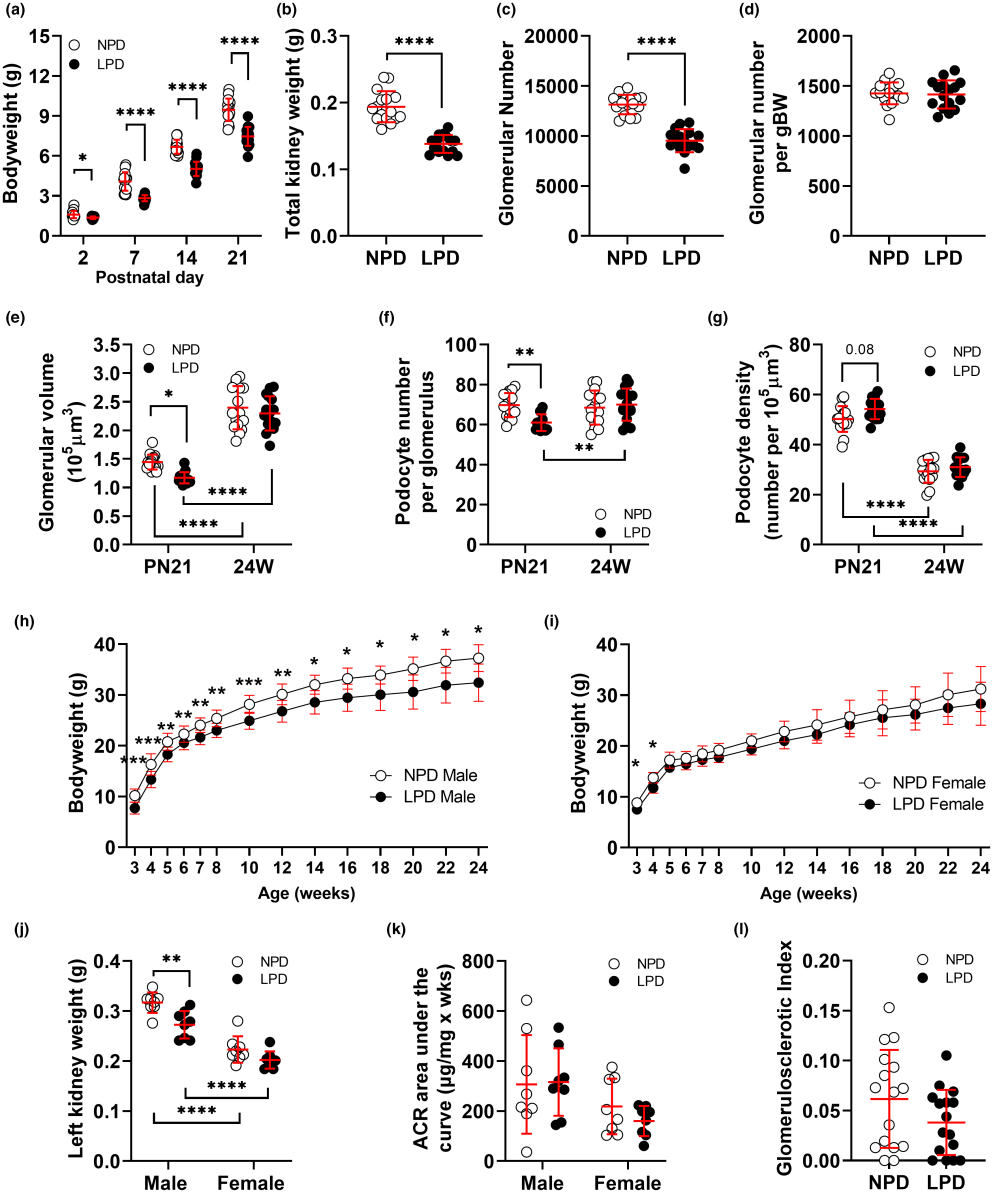
LPD induces growth restriction, low nephron endowment, and low podocyte endowment at weaning, but normal kidney function and histology at 24 weeks of age. (a) Pre‐weaning bodyweight (P2‐21); data analyzed by repeated measures 3‐way ANOVA for diet, sex, and time; no sex difference identified so data were consolidated and analyzed by 2‐way ANOVA for diet and time. (b) Total kidney weight (left and right kidney combined), (c) total glomerular number per kidney and (d) glomerular number per gram of bodyweight at P21; data analyzed by 2‐way ANOVA for diet and sex, no sex difference identified (e) glomerular volume, (f) podocyte number per glomerulus, and G) podocyte density at P21 and 24 weeks of age; data analyzed by three‐way ANOVA for diet, sex, and time, no sex difference identified so data were consolidated and analyzed by 2‐way ANOVA for diet and time. (h, i) Bodyweight from P21 to 24 weeks of age in male (h) and female (i) offspring; presented separately to show the identified sex difference following repeated measures 3‐way ANOVA for diet, sex, and time. (j) Left kidney weight and (k) albumin: creatinine area under the curve at 24 weeks in male and female offspring indicating the sex difference identified following 2‐way ANOVA for diet and sex. (l) glomerulosclerotic index in NPD and LPD offspring at 24 weeks of age; data analyzed by 2‐way ANOVA for diet and sex, no sex difference identified. NPD (white circles), LPD (black circles). Asterisks above data points represent significant differences between NPD and LPD; asterisks below datapoints represent significant differences between P21 and 24 weeks. In all cases, values are mean ± SD. where *p < 0.05, **p < 0.01, ***p < 0.001 and ****p < 0.0001 following multiple comparisons. P, postnatal day; 24 W, 24 weeks.

At P2, LPD offspring weighed 16% less than NPD offspring (*p* = 0.02). This weight differential increased to 36% at P7, 28% at P14, and 24% at P21 (*p* < 0.0001 at each timepoint; Figure 4a; Table 2). At P21, total kidney weight in LPD was 34% less than in NPD offspring (*p* < 0.0001; Figure 4b) and glomerular number was 32% lower (NPD 13141 ± 243 glomeruli per kidney, LPD 9528 ± 288 glomeruli per kidney, *p* < 0.0001; Figure 4c). Glomerular number/gBW was similar in LPD and NPD offspring (Figure 4d).

### 3.2 Maternal LPD offspring have low podocyte endowment at P21

Glomerular and podocyte indices were similar in male and female offspring at P21. On average, LPD glomeruli were 20% smaller than NPD glomeruli (Figure 4e; *p* < 0.05) and contained 13% fewer podocytes (Figure 4f; Table 2; *p* < 0.01). NPD glomeruli contained 69.7 ± 1.5 podocytes whereas LPD glomeruli contained 61.0 ± 1.0 podocytes. Podocyte density was 8% higher in LPD glomeruli than NPD glomeruli, but this difference was not statistically significant (Figure 4g; *p* = 0.08).

### 3.3 Maternal LPD offspring weaned onto standard chow underwent significant catch‐up growth

Growth trajectories for male and female LPD and NPD offspring throughout the experimental period are shown in Figure 4h,i. Male LPD offspring weighed 26% less than male NPD offspring at P21 (weaning) (Figure 4h). LPD males underwent significant catch‐up growth in the early weaning period, so that by 5 weeks of age LPD males weighed just 13% less than NPD males. This weight differential persisted throughout the remainder of the experimental period. In line with the reduced bodyweight, kidney weight in LPD male offspring remained 15% lower than NPD male offspring (Figure 4j). In contrast, female LPD offspring weighed 16% less than female NPD offspring at weaning (Figure 4i). By 5 weeks of age, NPD and LPD females had similar bodyweights, and this was the case for the remainder of the experimental period (Figure 4i), indicating complete catch‐up growth in female offspring. Similarly, kidney weight in LPD female offspring was similar to NPD female offspring at 24 weeks (Figure 4j).

### 3.4 Glomerular volume and podocyte number is normal in maternal LPD offspring at 24 weeks of age

Similar to P21, glomerular and podocyte indices at 24 weeks were similar in male and female offspring. As expected, glomerular volume increased significantly between P21 and 24 weeks in both NPD and LPD offspring (Figure 4e). The lower glomerular volume observed in LPD offspring at P21 was no longer apparent at 24 weeks (Figure 4e). Interestingly, podocyte number per glomerulus in LPD offspring increased significantly between P21 and 24 weeks in LPD offspring (P21, 61.0 ± 1.0 podocytes per glomerulus; 24 weeks, 70.0 ± 2.0 podocytes per glomerulus; *p* < 0.01), a finding not observed in NPD offspring. As a result, podocyte number per glomerulus at 24 weeks was similar in NPD and LPD offspring (Figure 4f). As expected, with similar glomerular volumes and podocyte numbers at 24 weeks in NPD and LPD offspring, their podocyte densities were similar (Figure 4g).

### 3.5 Maternal LPD offspring weaned onto standard chow have normal albumin excretion and renal histology at 24 weeks of age

Urinary albumin excretion in females was significantly lower than in males at 24 weeks (*p*_sex_ = 0.02; Figure 4k). Albumin excretion in male and female LPD offspring between 14 and 24 weeks of age was within the normal range **(** *p*_diet_ = 0.61; Figure 4k). At 24 weeks of age, GSI was similar in male and female offspring (*p*_sex_ = 0.30). NPD and LPD offspring had a similar glomerulosclerotic index at 24 weeks (*p*_diet_ = 0.11; Figure 4l).

### 3.6 Induction of diabetes in LPD and NPD offspring

**FIGURE 5:**
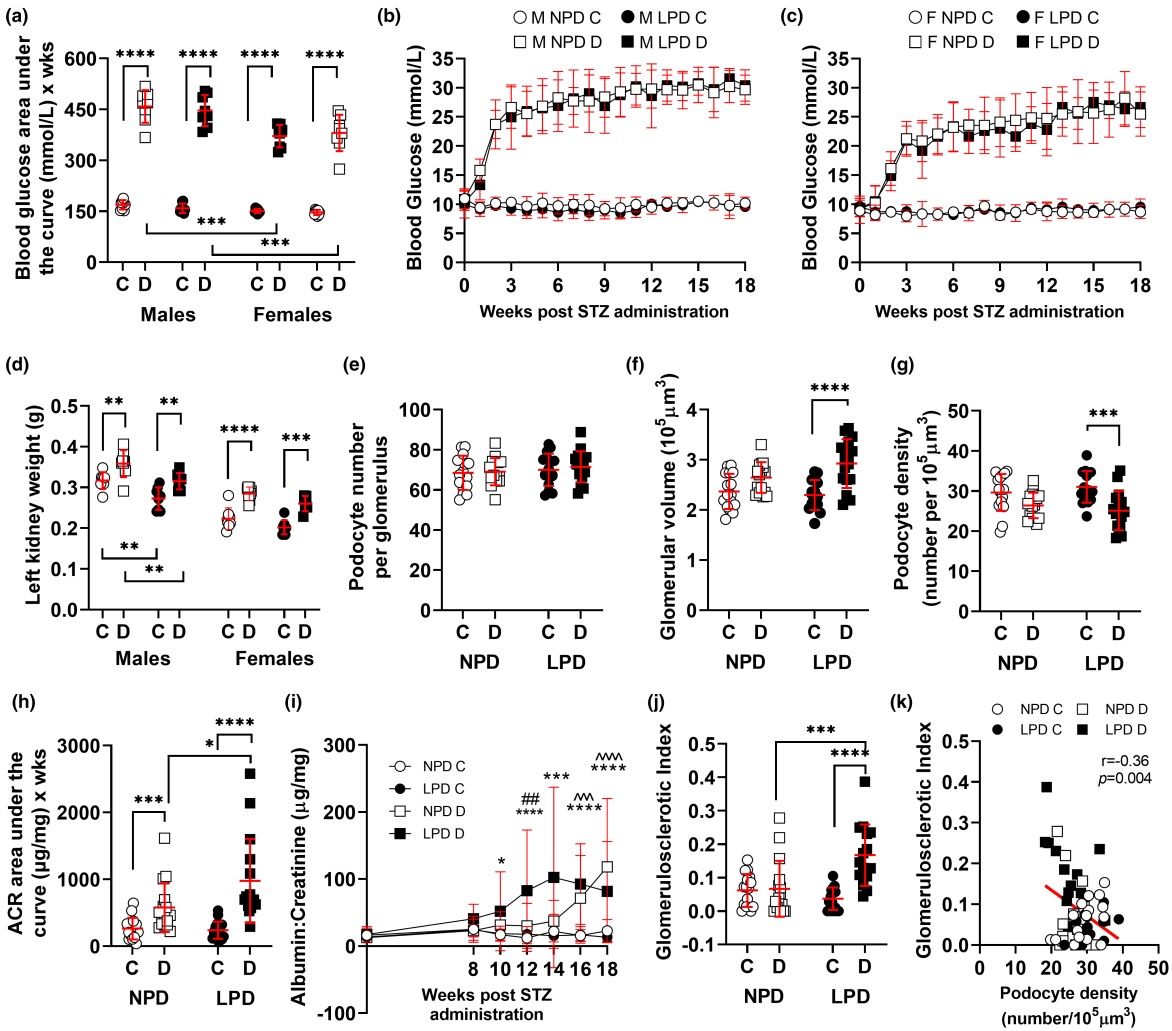
Disease susceptibility following induction of diabetes. (a) Blood glucose level presented as area under the curve (AUC) (analyzed by 3‐way ANOVA for diet, sex, and treatment) showing the significant difference between males and females. Blood glucose in male (b) and female (c) offspring across the 18‐week experimental period analyzed by 3‐way ANOVA with repeated measures for diet, treatment, and time. (d) kidney weight at 24 weeks following 18 weeks of hyperglycemia; analyzed by three‐way ANOVA for diet, sex, and treatment. Values are mean ± SD, where **p < 0.01, ***p < 0.001, ****p < 0.0001 following multiple comparisons, differences between sexes not shown. c, control (citrate buffer); d, diabetic (STZ treatment). (e) Podocyte number per glomerulus, (f) glomerular volume, (g) podocyte density, and (h) albumin: creatinine area under the curve, in control and diabetic NPD and LPD offspring. Each dataset in e–h analyzed by three‐way ANOVA for diet, sex, and treatment. No sex difference was identified, data were consolidated and analyzed by 2‐way ANOVA for diet and treatment; in all cases values are mean ± SD, where *p < 0.05, ***p < 0.001 and ****p < 0.0001 following multiple comparisons. (i) Albumin: creatinine across the experimental period; no sex difference identified in ACR AUC, so ACR analyzed by repeated measures 3‐way ANOVA for diet, treatment, and time where *p < 0.05 between LPD control and LPD diabetic, ***p < 0.001 between LPD control and LPD diabetic, ****p < 0.0001 between LPD control and LPD diabetic, ^^^*p* < 0.001 between NPD control and NPD diabetic, ^^^^*p* < 0.0001 between NPD control and NPD diabetic, ##*p* < 0.01 between NPD diabetic and LPD diabetic offspring. (j) Glomerulosclerotic index at 24 weeks of age in control and diabetic NPD and LPD offspring, data analyzed by three‐way ANOVA for diet, sex, and treatment, no sex difference was identified, data were consolidated and analyzed by 2‐way ANOVA for diet and treatment with values presented as mean ± SD, where ***p < 0.001 and ****p < 0.0001 following multiple comparisons. (k) Linear regression of the relationship between podocyte density and glomerulosclerotic index at 24 weeks of age (18 weeks of hyperglycemia in diabetic animals). In a–k: NPD—control (white circles), LPD—control (black circles), NPD—diabetic (white squares), LPD—diabetic (black squares). C, control; d, diabetic; M, male; f, female.

Diabetes was induced in male and female LPD and NPD offspring at 6 weeks of age, with 5 hour fasting glucose measured in control and diabetic mice weekly for a further 18 weeks (24 weeks of age). Calculation of blood glucose area under the curve (AUC) showed that blood glucose profiles in control NPD and control LPD were similar and the same for both sexes (Figure 5A). STZ treatment significantly increased blood glucose (*p*_treatment_ = <0.0001), with the 2.8‐fold increase in males significantly greater than the 2.5‐fold increase in females (*p*_treatment*sex_ = <0.001). The effect of STZ treatment on blood glucose AUC was similar in maternal NPD and LPD offspring (*p*_treatment*diet_ = 0.63; Figure 5a). We also assessed glucose profiles across the experimental period in males (Figure 5b) and females (Figure 5c), identifying there was no difference in blood glucose between diabetic NPD and diabetic LPD animals at any timepoint.

### 3.7 Diabetes increased glomerular volume in maternal LPD offspring resulting in low podocyte density at 24  weeks

Kidney weight at 24 weeks was significantly lower in females than males (*p*_sex=_ < 0.0001) and kidney weight was higher in diabetic than control mice in all groups (*p*_treatment_ = < 0.0001; Figure 5d). Glomerular and podocyte indices after 18 weeks of diabetes (24 weeks of age) were similar in male and female offspring. Overall, podocyte number was unaffected by 18 weeks of hyperglycemia (*p*_treatment_ = 0.61; Figure 5e). Glomerular volume was 24% greater in LPD diabetic offspring than in LPD control offspring (*p* < 0.0001). There was a trend for a similar, albeit lower (12%) increase in glomerular volume in NPD diabetic offspring (*p* = 0.07; Figure 5f).

The increase in glomerular volume in diabetic LPD offspring was associated with a 20% lower podocyte density than in control LPD offspring (*p* < 0.001; Figure 5g). There was a trend for a similar (11%) lower podocyte density in NPD diabetic compared with NPD control offspring (*p* = 0.07; Figure 5g).

### 3.8 Low podocyte density in maternal LPD diabetic offspring is associated with increased albumin excretion and glomerulosclerotic index

There was no statistical difference between male and female offspring for albumin excretion (AUC) or GSI after 18 weeks of diabetes (24 weeks of age). Diabetic LPD offspring demonstrated a 3.7‐fold greater increase in albumin excretion AUC (*p* < 0.0001) than control LPD offspring (Figure 5h). Albumin excretion AUC in diabetic NPD offspring was 2.2‐fold greater than in control NPD offspring (*p* < 0.001). Albumin excretion AUC was significantly higher in diabetic LPD offspring than diabetic NPD offspring following 18 weeks of hyperglycemia (*p* < 0.05; Figure 5h).

We also assessed albumin excretion at individual timepoints across the experimental period (Figure 5i). Assessment of albumin excretion at 8, 10, 12, 14, 16, and 18 weeks post‐STZ administration showed that diabetic NPD offspring were microalbuminuric at 16 and 18 weeks post‐STZ while NPD control offspring were not (*p* < 0.001 at 16 weeks, *p* < 0.0001 at 18 weeks; Figure 5i). Diabetic LPD offspring were microalbuminuric from 10 weeks (p < 0.05) to the completion of the study at 18 weeks (*p* < 0.0001 at 12, 14, 16, and 18 weeks; Figure 5i) while control LPD offspring were not. Albumin excretion was significantly higher in diabetic LPD offspring than diabetic NPD offspring at 12 weeks (Figure 5i; *p* < 0.01) but then was similar in these two groups until the conclusion of the study.

Diabetic LPD offspring showed significantly greater GSI than control LPD offspring (*p* < 0.0001; Figure 5j), a finding not observed in NPD offspring (*p* = 0.97). GSI was significantly higher in diabetic LPD offspring than diabetic NPD offspring following 18 weeks of hyperglycemia (*p* < 0.001; Figure 5j). As expected, GSI was inversely correlated with podocyte density (*p* = 0.004; *r* = −0.36; Figure 5k) indicating that the relative podocyte depletion observed in diabetic mice (particularly LPD diabetic mice) is strongly linked to the development of glomerulosclerosis.

## 4 DISCUSSION

The major findings from this study were 1) podocyte number per glomerulus in LPD offspring at weaning was lower than in NPD offspring, but normalized by 24 weeks of age; 2) at 24 weeks, LPD offspring with a 32% nephron deficit but normalized podocyte number did not show increased susceptibility to albuminuria or glomerulosclerosis; 3) hyperglycemia did not alter podocyte number in NPD or LPD offspring; 4) hyperglycemia‐induced increased glomerular volume and lower podocyte density (relative podocyte depletion), with LPD offspring more susceptible than NPD offspring; 5) LPD offspring developed albuminuria earlier than NPD offspring and developed glomerulosclerosis. These findings are discussed in turn.

### 4.1 Podocyte number per glomerulus in LPD offspring at weaning was lower than in NPD offspring but normalized by 24 weeks of age

Podocyte number per glomerulus in both male and female LPD offspring was 10–15% lower than in NPD offspring at 3 weeks of age. To our knowledge, this is just the third report of developmental programming of low podocyte endowment. Previously, Goncalves et al. (2020) reported that prenatal exposure of pregnant mice to hypoxia resulted in a programmed podocyte deficit in male offspring at 3 weeks of age of approximately 15%, with hypoxic mice having 53 podocytes per glomerulus compared with 63 in control normoxic offspring. In contrast, hypoxia did not give rise to low podocyte endowment in female offspring (Goncalves et al., 2020). Cullen‐McEwen et al. (2021) reported that a maternal low protein diet resulted in a podocyte deficit of 15% in male Sprague–Dawley rat offspring (female offspring were not studied) at 3 weeks of age. These findings of podocyte deficits in the present study and these two earlier studies in two species and two models of developmental programming suggest that other perturbations to the maternal environment likely result in a podocyte deficit. It is well established that a range of perturbations to the maternal environment including low protein diet (Hoppe, Evans, Bertram, et al., 2007; Langley‐Evans et al., 1999; Zimanyi et al., 2004), hypoxia (Walton et al., 2017), alcohol exposure (Gray et al., 2008, 2010; Kenna et al., 2012), gestational diabetes (Hokke et al., 2016), and glucocorticoid exposure (Dickinson et al., 2007; Singh et al., 2007; Wintour et al., 2003) result in a nephron deficit and this may well be the case also for podocyte endowment. Given the association between podocyte depletion and glomerular pathology (Puelles et al., 2019; Puelles, van der Wolde, et al., 2016; van der Wolde et al., 2022; Wharram et al., 2005; Wiggins et al., 2005) further studies on the regulation of podocyte endowment are warranted. Indeed, low podocyte endowment may be an important factor leading to the increased risk of CKD for patients born small or early (Luyckx et al., 2013, 2017; White et al., 2009). The consistency of the extent of the podocyte deficit in the three studies, namely 10–15%, is remarkable and also warrants further investigation, as do the timing and underlying mechanisms leading to the podocyte deficits.

Interestingly, podocyte number per glomerulus in LPD offspring in the present study increased significantly in both male and female offspring between 3 and 24 weeks of age, by approximately 9 podocytes per glomerulus (a 13% increase). As a result, podocyte number per glomerulus at 24 weeks was similar in NPD and LPD offspring. Olivetti et al. (1980) previously described a similar pattern between young, adult, and uninephrectomized rats. Interestingly, Cullen‐McEwen et al. (2021) recently reported a similar postnatal increase in podocyte number in male rats fed a maternal low protein diet before birth and then switched to a normal diet. They found that between postnatal 3 weeks and 6 months of age, podocyte number per glomerulus increased by approximately 9% (12 podocytes per glomerulus), although podocyte number remained 9% lower than NPD control rats. While podocytes have traditionally been described as post‐mitotic and terminally differentiated and incapable of undergoing mitosis, these findings indicate that between weaning and 6 months of age rat and mouse glomeruli with a low protein diet‐induced podocyte deficit are able to increase their podocyte endowment. Unfortunately, our experimental design did not allow the determination of when these additional podocytes appeared, but we expect that this increase in podocytogenesis may be tightly regulated with catch‐up growth post weaning (between 3 and 5 weeks of age) and that podocyte number may have been similar to controls by 6 weeks of age when STZ was administered. Whether this podocyte gain represents a delay and/or extension of the period of podocytogenesis is unclear. Interestingly, in the study of Cullen‐McEwen et al. (2021) in rats mentioned above, the podocyte gain in LPD offspring between P21 and 6 months occurred in outer glomeruli rather than inner glomeruli. Given that outer glomeruli are the last to form during nephrogenesis, this suggests that podocytogenesis was not complete in these outer glomeruli at 3 weeks of postnatal life. Additionally, the origin of these new podocytes remains unclear. They may represent late‐maturing podocytes that were not present at P21 or did not express the nuclear marker (p57) we used to identify post‐mitotic podocytes. Alternatively, these new podocytes may have been derived from parietal epithelial cells (Lasagni & Romagnani, 2010; Shankland et al., 2013) or cells or renin lineage (Kaverina et al., 2020; Pippin et al., 2014; Shankland et al., 2017).

Few studies have compared podocyte number in children with that in adults. However, in a study of Caucasian Americans without overt kidney disease, Puelles et al. (2015) found that children aged under 3 years contained 452 podocytes per glomerulus whereas adults contained 558 podocytes per glomerulus. Despite the fact that only four children (≤3 years old) and 12 adults were studied, the findings illustrated that this difference in podocyte number was largely driven by the highest podocyte count in the largest adult glomeruli. Again, future studies investigating the duration of podocytogenesis, and origin of postnatal podocytes are warranted.

### 4.2 At 24 weeks LPD offspring with a 32% nephron deficit but normal podocyte number did not show increased susceptibility to albuminuria or glomerulosclerosis

Despite a 32% deficit in nephron endowment and significant catch‐up growth, LPD offspring had normal albumin excretion and renal morphology at 6 months of age. This result was somewhat unexpected given the established link between low nephron endowment and risk of renal disease in adulthood (Luyckx et al., 2013, 2019; Luyckx & Chevalier, 2022). The thrifty phenotype hypothesis proposes that in conditions of undernutrition a fetus responds by limiting growth, favoring a phenotype that will most likely thrive in the anticipated postnatal environment of nutrient scarcity (Bar et al., 2021). However, if offspring born small undergo excessive postnatal catch‐up growth the kidneys are placed at greater risk because they do not have an adequate renal reserve to cope with the increased functional load. While catch‐up growth did occur in LPD mice their bodyweight at 6 months was not excessive, with LPD males still weighing 12% less than NPD males, and LPD and NPD females having similar bodyweights. Whether levels of albumin excretion are maintained as the offspring age is not known. Similar to our study, Lim et al. (2011) found in Kyoto rats that maternal LPD induced a low nephron number and a similar level of catch‐up growth to that observed in the current study yet maintained normal albumin excretion (in non‐diabetic animals). In contrast (Menendez‐Castro et al., 2013) induced offspring in utero growth restriction (maternal LPD during pregnancy) and while they also showed offspring catch‐up growth and a nephron deficit, rats presented with significant albuminuria at 70 days of age (males only, females not studied). Menendez‐Castro also utilized electron microscopy to identify pathological podocyte alterations, including podocyte enlargement and foot process effacement, both of which were independent of any changes in blood pressure. In addition, they showed altered expression of WT1 + KTS and ‐KTS isoforms from birth and concluded that growth restriction results in podocyte damage due to dysregulation of WT1 and that imbalance of WT1 isoforms disturbs nephrogenesis and reduces the ability to maintain podocyte integrity, rendering IUGR rats more susceptible to renal disease. Whether we would also see podocyte alterations at the electron microscopy level is unknown but would be of interest.

### 4.3 Hyperglycemia did not alter podocyte number in NPD or LPD offspring

Podocyte loss is a well‐known feature of human type 2 diabetes. Pagtalunan et al. (1997) reported that reduced podocyte number correlated with albuminuria and loss of glomerular filtration rate in Pima Indians with type 2 diabetes. Subsequent human studies confirmed these findings and suggested that podocyte loss and the resultant reduction in podocyte density contributed to the progression of diabetic nephropathy (Dalla Vestra et al., 2003; Lemley et al., 2000; Meyer et al., 1999; Weil et al., 2012; White & Bilous, 2004). Links between podocyte loss and disease progression in type 1 diabetes are less clear (Harindhanavudhi et al., 2015; Steffes et al., 2001; Toyoda et al., 2007; White et al., 2002). Steffes et al. (2001) reported that podocyte number decreased in patients with a relatively short duration of type 1 diabetes. In contrast, White et al. (2002) found no difference in podocyte number between controls and patients with type 1 diabetes.

In the present study, 18 weeks of STZ‐induced hyperglycemia did not result in any changes in podocyte number in either NPD or LPD offspring of either sex. This is surprising given that podocyte injury and loss have been previously reported in studies of STZ‐induced hyperglycemia, although the extent of the reported podocyte loss varies considerably between studies. For example, Gao et al. (2016) reported a greater than 50% reduction in podocyte number per glomerular cross‐section 8 weeks after STZ‐induced moderate diabetes in male CD‐1 mice. In contrast, Siu et al. (2006) reported a mild 15% reduction in podocyte number per glomerular cross‐section in moderately diabetic male Wistar rats and C57BL/6J mice, while Gross et al. (2003) reported a 38% loss in podocyte number 6 months after STZ induction of severe diabetes in male Sprague Dawley rats. It is important to note that these differing outcomes in podocyte number likely reflect differences between studies in the species and strain studied, the level and period of hyperglycemia, and the methodology used to count podocytes. The present study differs from previous reports in that the level of hyperglycemia and glomerular pathology (see below) were mild, and podocytes were counted in whole glomeruli, not in sectioned glomeruli (glomerular profiles or cross‐sections). This counting approach provides the absolute number of podocytes per glomerulus without any influence of well‐known confounding factors such as section thickness, tissue shrinkage, and glomerular volume.

### 4.4 Hyperglycemia induced increased glomerular volume and lower relative podocyte density, with LPD offspring more susceptible than NPD offspring

While STZ‐induced hyperglycemia was not associated with podocyte loss at 18 weeks, it was associated with glomerular hypertrophy, particularly in LPD offspring. As a result, podocyte density was 20% lower in LPD diabetic offspring than in LPD control offspring. A similar trend was found between control and diabetic NPD offspring (12% increase in glomerular volume and 11% decrease in podocyte density) although this did not reach statistical significance (*p* = 0.07 in both cases). Glomerular hypertrophy and an associated decrease in podocyte density have been reported in human and animal studies of diabetic kidney disease (Siu et al., 2006; White et al., 2002). Moreover, decreased podocyte density, a form of podocyte depletion is associated with development of albuminuria and glomerulosclerosis in non‐diabetic models of glomerular pathology (Puelles et al., 2019; Puelles, Cullen‐McEwen, et al., 2016; Puelles, van der Wolde, et al., 2016; Wharram et al., 2005; Wiggins et al., 2005). These findings were expected as many studies have reported increased glomerular volume in diabetic animals (Gross et al., 2003, 2004; Ichikawa et al., 2020; Jones et al., 2006; Kiran et al., 2012; Rasch, 1979; Satoh et al., 2010). The interesting finding here is that hyperglycemia drove a 24% increase in glomerular hypertrophy (and a corresponding decrease in podocyte density) in LPD offspring, twice that observed in NPD offspring, indicating the low nephron endowment impacted the risk of disease. Hyperfiltration, is a well characterized consequence of early diabetes. While this compensatory adaptive response can help maintain renal homeostasis, the associated increase in glomerular capillary pressure damages many glomeruli, causing hypertrophy in residual glomeruli and a cycle of renal damage. In this study, hyperfiltration was superimposed on two degrees of filtration capacity, those with normal (NPD) and those with low nephron endowment (LPD). The lower filtration capacity in LPD offspring presumably led to earlier/greater glomerular hyperfiltration and hypertrophy and thereby significant renal diabetic injury. There is a multitude of evidence that a series of “hits” can explain the risk of adverse health outcomes across a lifetime (McMullen & Mostyn, 2009). In this study, the first hit, a reduced nephron endowment appears to have increased the vulnerability to a second “hit” of hyperglycemia resulting in greater glomerular hypertrophy in the attempt to maintain kidney function. Other studies have shown similar findings, with Østergaard et al. (2020) recently describing the additive effects of genetic diabetes and unilateral nephrectomy on glomerular hypertrophy. Similar to the present study Jones et al. (2006), observed growth restriction and a nephron deficit in offspring following maternal protein restriction during pregnancy. The induction of diabetes in these rat offspring increased glomerular volume in both NPD and LPD offspring and while podocyte density was lowest in LPD diabetic animals this difference did not reach statistical significance (Jones et al., 2006). This latter finding may suggest low podocyte endowment in the LPD offspring, although this was not assessed. Furthermore, the area of basement membrane covered by each podocyte was greater in LPD diabetic animals than both LPD controls and NPD diabetic animals and correlated with albumin excretion (Jones et al., 2006) supporting the concept that the altered renal development as a result of maternal protein restriction impacts the response to a secondary stimulus.

The 12–24% increase in glomerular volume in diabetic mice observed in the present study is relatively mild compared with the ~40 to >100% increases reported in previous animal studies (Gross et al., 2003, 2004; Ichikawa et al., 2020; Kiran et al., 2012; Rasch, 1979; Satoh et al., 2010). This difference likely lies in the genetic background of the mice used, as C57BL/6 mice are notorious for resistance to developing renal injury in experimental models of kidney diseases, including diabetic nephropathy (Kitada et al., 2016).

### 4.5 LPD offspring developed albuminuria earlier than NPD offspring and also developed glomerulosclerosis

In the present study, the decreased podocyte density in LPD diabetic offspring was associated with increased albumin excretion and glomerulosclerosis. Consistent with the mild increase in glomerular volume and decrease in podocyte density, NPD offspring showed a trend for a mild increase in albumin excretion but no increase in glomerulosclerosis. These findings support the concept that low nephron endowment impacts the susceptibility to renal disease in this model. To our knowledge, only two previous studies have utilized a two‐hit model to study the renal outcomes of a maternal LPD (with growth restriction and nephron number) followed by STZ induction of hyperglycemia in offspring (Jones et al., 2001; Lim et al., 2011). Similar to this study, Jones et al. (2001) found no alteration in urinary albumin excretion in non‐diabetic LPD compared to non‐diabetic NPD rats. Interestingly, 1 week of diabetes increased albumin excretion in both diabetic NPD and diabetic LPD offspring although there was no difference in the degree of albuminuria between groups (Jones et al., 2001). Lim et al. (2011) also found that the renal functional response to 8 weeks of hyperglycemia was similar in LPD and NPD offspring. Interestingly, the albumin excretion in the Lim et al. (2011) study was greater in diabetic LPD than diabetic NPD offspring although it did not reach statistical significance, leading the authors to conclude that over a greater period of time the renal dysfunction associated with hyperglycemia may have been heightened in LPD offspring (Lim et al., 2011). Our differing finding of increased susceptibility to diabetes‐induced albumin excretion and glomerulosclerosis is likely associated with the length of time the LPD mice were exposed to hyperglycemia (18 weeks in the current study vs 8 weeks in the Lim et al. (2011) study) given our study identified mild albuminuria only at 10+ weeks in diabetic LPD mice and 14+ weeks in diabetic NPD mice. In addition, our finding that diabetic LPD offspring developed albuminuria 4 weeks before diabetic NPD offspring supports the two “hit” hypothesis that LPD offspring were more vulnerable to develop albuminuria than NPD offspring, the result of a predisposition to the secondary insult of hyperglycemia.

### 4.6 Limitations

While non‐diabetic LPD offspring showed no evidence of increased susceptibility to their growth restriction and low nephron endowment at 6 months of age. Given the association between intrauterine growth restriction and glomerular hyperfiltration and hypertension measurements of glomerular filtration rate (GFR; as a marker of glomerular function) and blood pressure would have provided additional insight into the susceptibility to kidney dysfunction in LPD offspring. It would also have been interesting to examine these offspring at later timepoints to observe their susceptibility to adverse renal outcomes with increasing age.

LPD‐diabetic mice showed only mild increases in albumin excretion and glomerular pathology despite having severe hyperglycemia over 18 weeks. Again, GFR and/or blood pressure measurements would have been beneficial to further understand the impact of hyperglycemia on kidney function in growth‐restricted offspring. Perhaps the mild changes observed are associated with the strain, as C57BL/6 mice are renowned for resistance to development of diabetic kidney disease and kidney failure. Potentially, an additional stressor, such as uninephrectomy, may have revealed an underlying susceptibility to disease in these mice.

Hyperglycemia in this study is a model of type 1 diabetes recapitulating the destruction of pancreatic beta cells. Whether the same findings would have been observed in models of type 2 diabetes are worth investigation.

We utilized antibodies rather than a transgenic marker to label and count podocytes in whole glomeruli. Whether STZ administration or hyperglycemia altered the expression of p57 or synaptopodin used to identify podocytes is unknown, but in our view, this is unlikely.

## 5 CONCLUSIONS

The results of this study demonstrate that a permanent nephron deficit due to intrauterine growth retardation can increase the risk of albuminuria and glomerulosclerosis in a model of diabetes. These changes occurred in conjunction with a decrease in podocyte density but not podocyte number per glomerulus.

## AUTHOR CONTRIBUTIONS

All authors participated in review of the manuscript and the data shown and have read and approved the final version of the manuscript. LAC‐M and JFB specifically contributed to conception and design and also contributed to data analysis and data interpretation. LAC‐M, SG, JvDW, and KH contributed to data acquisition. LAC‐M, SG, and JFB prepared the manuscript. All authors critically reviewed the manuscript.

## FUNDING INFORMATION

No funding information provided.

## CONFLICT OF INTEREST

None to declare.

## ETHICS STATEMENT

All experiments were conducted in accordance with guidelines set by the Monash University Animals Ethics Committee (ethics approval number: MARP/2016/162).
